# Differential evolution of anti-VAR2CSA- IgG3 in primigravidae and multigravidae pregnant women infected by *Plasmodium falciparum*

**DOI:** 10.1186/1475-2875-7-10

**Published:** 2008-01-11

**Authors:** Juliette Guitard, Gilles Cottrell, Nellie Moulopo Magnouha, Ali Salanti, Tengfei Li, Sokhna Sow, Philippe Deloron, Nicaise Tuikue Ndam

**Affiliations:** 1 Institut de Recherche pour le Développement (IRD), UR010, Mother and Child Health in the Tropics, Université Paris Descartes, France; 2 Laboratoire de Parasitologie, APHP, Hôpital Bichat, Paris, France; 3 Centre for Medical Parasitology, University of Copenhagen, Copenhagen, Denmark; 4 Thiadiaye Hospital, Senegal

## Abstract

**Background:**

Pregnant women develop protective anti-VSA IgG1 and IgG3 when infected by *Plasmodium falciparum*. The major target of IgG from serum of infected pregnant women is VAR2CSA.

**Methods:**

In this study, ELISA was used to compare the level of VAR2CSA DBL5ε- specific IgG subclasses at enrolment and at delivery in a cohort of pregnant women in Senegal. All antibody measures were analysed in relation to placental infection according to parity.

**Results:**

The results show an interaction between immune response to placental malaria and parity. A higher level of anti- DBL5ε- IgG3 at enrolment and a higher increase between enrolment and delivery were found in primigravidae who presented with uninfected placenta at delivery in comparison to those who presented with an infection of the placenta. However, high antibody level at delivery was associated with the infection of the placenta in multigravidae.

**Conclusion:**

This high level of IgG3 in uninfected primigravidae suggests a protective role of these antibodies in this susceptible group, highlighting the importance of VAR2CSA in general and of some of its variants still to be defined, in the induction of protective immunity to pregnancy malaria.

## Background

*Plasmodium falciparum*-infected erythrocytes (IE) are able to bind various host receptors via the expression of variant surface antigens (VSAs) on the erythrocyte surface. *Plasmodium falciparum *erythrocyte membrane protein 1 (PfEMP1) is a VSA located on the IE surface, which undergoes clonal antigenic variation. Acquired protection against malaria is mediated, at least partially, by IgG targeting PfEMP1 [1]. Although this antibody response may directly inhibit IE adhesion to endothelial cells, it also might be implicated in opsonization. IgG1 and IgG3 are responsible for pathogen clearance via opsonization, sensitization of NK cells, and/or activation of the complement system [2]. A few studies have examined the pattern of IgG subtypes in the antimalarial response and have underlined the role of the anti-VSA cytophilic IgG (IgG1 and IgG3) in protection from malaria [3-5]. *In vitro*, these IgG mediate the phagocytosis of IE [6], a mechanism that may also play a major role in parasite elimination in humans.

Pregnancy-associated malaria (PAM) results in infant low birth weight, and maternal anaemia. IE accumulate in the placental intervillous space and bind to chondroitin sulfate A (CSA) via a specific PfEMP1 variant, VAR2CSA [7]. This protein is comprised of six Duffy binding like (DBL) domains, several of which (including DBL2X, DBL3X, DBL5ε-, DBL6ε-) have been shown to bind to CSA [8,9]. After successive pregnancies, women develop protective IgG antibodies against placental parasites. Serum from pregnant women from different geographical areas are able to recognize the surface of IE from pregnant women, suggesting that the antigenic target must be relatively conserved. Anti-VAR2CSA antibody levels correlate with the decrease of the rates of placental infection, low birth weight and maternal anaemia, and with the ability of the serum to inhibit the adhesion of IE to CSA (reviewed in [1]).

Two studies [10,11] demonstrate that anti-VSA IgG1 and IgG3 are the main antibody subclasses implicated in the anti-PAM response. Levels of IgG1 and IgG3 correlate with serum ability to inhibit the adhesion of IE to CSA *in vitro*. Larger studies are needed to determine if IgG1 and IgG3 are protective in pregnant women and to determine their target. As *var2csa *is over-expressed in placental parasites, and as anti-VAR2CSA IgG inhibit the IE adhesion to CSA and are associated with protection from PAM [12,13], whether or not malaria infection during pregnancy is able to induce VAR2CSA specific IgG1 and IgG3 was further examined. The level of DBL5ε- specific antibodies at enrolment and at delivery was compared in a cohort of pregnant women in relation to placental infection according to parity.

## Methods

Pregnant women were enrolled in a cohort study between 30 July and 15 October 2001 in Thiadiaye, 130 km east from Dakar [13]. Briefly, women pregnant for less than 6 months were enrolled if they were not infected with malaria parasites at that time, declared not to have had malaria since being pregnant, and were likely to be exposed to infective mosquito bites during their pregnancy. A total of 306 pregnant women were followed by active and passive detection through monthly ANC visits and through weekly home visits until delivery. Women presenting with fever and a positive blood smear were given curative treatment with chloroquine, the first-line antimalarial drug in Senegal at that time. However, 55/111 malarial infections were symptomless and detected afterwards by active case survey. At delivery, peripheral and placental blood was investigated by microscopy for the presence of malaria parasites.

The DBL5ε- domain of VAR2CSA from 3D7 was produced in baculovirus-infected SF9 cells, as described [12-14]. Recombinant MSP1 (yPfMSP1–19) was used as a control. Optimal concentrations of each protein were coated in 96-well plates, and the different subtypes of specific IgG were measured by ELISA. Plates were coated with 1 μg/mL concentrations of antigen. Wells were incubated with 100 μL of human plasma at dilutions optimized for each measure (1:200 for total IgG, 1:100 for IgG1 and IgG3, 1:50 for IgG2 and IgG4), followed by horseradish peroxidase-conjugated anti-human IgG (1:15,000) for total IgG measures. For the remaining IgG subclasses, purified mouse monoclonal antibodies against human IgG1 (clone MH1013, Caltag laboratories, Burlingame, CA), IgG2 (clone: MH1022, Caltag laboratories, Burlingame, CA), IgG3 (clone: MH1032, Caltag laboratories, Burlingame, CA) or IgG4 (clone: MH1042, Caltag laboratories, Burlingame, CA) were used. All reagents were used at predetermined optimal dilutions.

The optical density (OD) was obtained by subtracting the average OD of duplicate wells from that of the corresponding blank wells. Values were converted into arbitrary units (AUs), as follows:

$$AU=100\times [\frac{\ln\;(OD\;test\;sample)-\ln\;(OD\;negative\;control)}{\ln\;(OD\;positive\;control)-\ln\;(OD\;negative\;control)}]$$

Serum from 13 young Senegalese children, 18 French adult men and 31 French pregnant women were included as controls. Antibody responders were defined as those having an antibody level (in AU) > 2SDs above the mean absorbance of the negative control.

Analyses were performed with each antibody sub-class (IgG1, IgG2, IgG3, IgG4) in women with one detected malarial infection or more during the follow-up. For each sub-class, three "antibody variables" were created: the level of antibody at enrolment, the level of antibody at delivery, and the variation between enrolment and delivery (level at delivery – level at enrolment). These three "antibody variables" were compared by Wilcoxon test between women presenting with a placental infection at delivery (positive placental blood smear) vs. those without placental infection. Analyses were stratified on parity (primigravidae and multigravidae).

As the onset of a febrile episode during the follow-up (and the subsequent treatment) is a potential confounder in this analysis, a "febrile episode" dummy variable has been created: existence or not of one or more febrile episodes during the follow-up. The relation between this variable and each of the three "antibody variables" was checked by Wilcoxon test (stratified on parity). When both "febrile episode" and "placental infection" variables showed significant difference of antibodies levels, they were simultaneously entered in a multivariate linear regression model.

## Results

### Study population

Among the 306 women, 12 were lost to follow-up, and at least one serum sample was lacking for 56 of them: antibody titres were measured in 261 women at enrolment, and in 240 at delivery. Among the 238 women having antibody measurements at both enrolment and delivery, 53 were primigravidae (22.3%) and 185 multigravidae (77.7%). The prevalence of placental infection (positive blood smear) was around 15% (35/238) at delivery. A total of 138 women did not present with a *P. falciparum *infection during the follow-up and had measures of antibody titres at enrolment and at delivery; the mean levels of IgG3 antibodies was unchanged, at 32.4 (sd = 37.7) and 34.7 (sd = 38.1) AU respectively. One hundred and eleven women presented with at least one *P. falciparum *infection during the follow-up, and measures of antibody titres at enrolment, 100 of these also had antibody measurement at delivery. Among these 111 women, 56 presented at least once during the follow-up with a malaria clinical attack requiring treatment. The other 55 presented with asymptomatic infections, and therefore did not receive antimalarial treatment.

### Seroreactivity of anti- DBL5ε- IgG

It has been shown that plasma level of VSA-specific IgG is dominated by IgG1. High levels of anti- DBL5ε- IgG1 and IgG3 were observed in the present cohort of senegalese pregnant women both at enrolment and at delivery.

**Figure 1:**
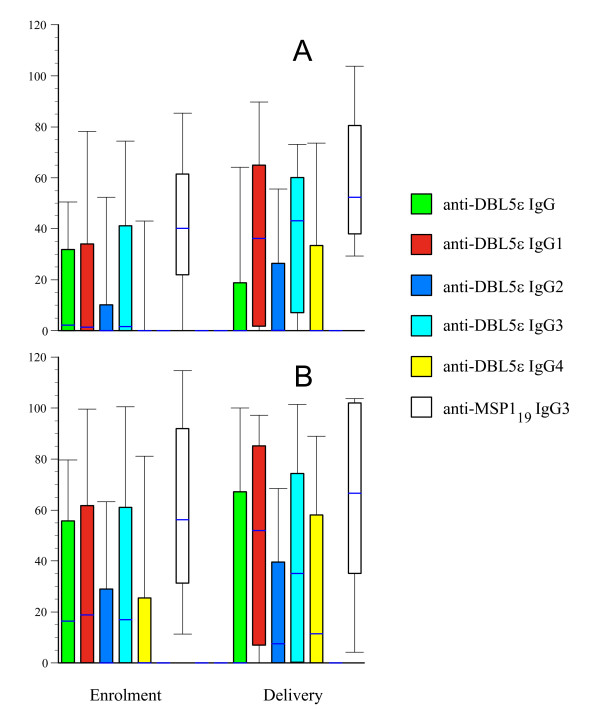
**Parity-dependency of the plasma antibody reactivity to VAR2CSA and MSP1_19 _recombinant proteins**. ELISA was carried out on DBL5ε- and MSP1_19 _coated plates. IgG, IgG1, IgG2, IgG3, and IgG4 directed against DBL5ε-, as well as against MSP1_19 _. Antibody plasma levels are expressed as arbitrary units (AU) and are shown at enrolment and at delivery for primigravid (panel A, n = 53) and multigravid women (panel B, n = 185) from Senegal. The top, bottom, and line through the middle of the box correspond to the 75^th ^percentile, 25^th ^percentile, and 50^th ^percentile (median), respectively. The whiskers on the bottom extend from the 10^th ^percentile and top 90^th ^percentile.

In a global analysis for all the women, all subclasses levels increased between enrolment and delivery (all p < 0.001) (Figure 1). The plasma levels of IgG anti-MSP1 did not vary significatively between enrolment and delivery (p = 0.13), as previously described for IgG directed against non PAM-VSAs [11]. Total IgG anti-DBL5ε- did not vary between enrolment and delivery in primigravidae and in multigravidae (p = 0.24 and 0.95 respectively), whereas all IgG subclasses levels significantly increased between enrolment and delivery in primigravidae as well as in multigravidae (all p < 0.02) excepted the IgG4 level that did not increase in primigravidae (p = 0.13).

The only subclass, that showed significant differences between women with infected and uninfected placenta at delivery, according to parity, was IgG3 and only the results concerning this subclass will, therefore, be detailed here.

### Serum IgG3 level to VAR2CSA DBL5ε-

Anti-DBL5ε- IgG3 levels in the 111 women infected during pregnancy and who presented with or without placental infection are shown in Table 1. The results show two opposite situations according to parity. In primigravidae, women without placental infection presented with higher IgG3 levels for the three "antibody variables" at enrolment, at delivery, and variation between enrolment and delivery (the difference at enrolment was only marginally significant, p < 0.1, probably due to a lack of power resulting from the small sample size), as compared to women with placental infection. This relation was not modified when the "febrile episode variable" was taken into account. On the contrary, in multigravidae higher levels of IgG3 were observed in women with infected placenta (only significant at delivery). The variation between enrolment and delivery was significant in the univariate analysis (p = 0.04), but not when the "febrile episode variable" was taken into account (p = 0.21). Neither other anti-DBL5ε- subclasses, nor anti-MSP1 IgG3 antibodies were related to placental infection when similar analyses were performed (all p > 0.10).

**Table 1:** Levels of IgG3 according to placental infection and parity

		Uninfected placenta		Infected placenta		*P *value **
			
		No	Mean (sd*)	No	Mean (sd)	
	Primigravidae	14	27.0 (33.9)	13	14.5 (29.0)	0.09
Enrolment	Multigravidae	67	36.8 (42.7)	17	42.7 (53.9)	0.54

	Primigravidae	13	64.3 (21.6)	12	39.2 (24.7)	0.03
Delivery	Multigravidae	59	45.7 (37.4)	16	73.4 (30.6)	0.01

Variation	Primigravidae	13	41.9 (24.9)	12	23.5 (29.3)	0.06
delivery – enrolment	Multigravidae	59	10.6 (40.2)	16	28.0 (63.8)	0.21

* sd, standard deviation

** after having taken into account the "febrile episode variable"

## Discussion

The aim of this study was to compare the acquisition of the different sub-classes of VAR2CSA-specific antibodies in primigravidae and multigravidae and to assess their role in protection from placental infection. Previously [13], the level of total IgG against recombinant DBL1X, DBL5ε- and DBL6ε- in the same cohort of women as determined at enrolment and at delivery showed that significant increases were associated with women who experienced at least one peripheral parasitemia during the survey. In the current study, the total IgG level was similar at both dates in women who never presented with a positive thick blood smear during their pregnancy. Furthermore, some (5/138) of these women presented with a placental infection, revealing an infection not detected during the follow-up. Thus, further analysis focussed towards women with one or more proven malarial infection during pregnancy.

IgG directed against the DBL5ε- domain seem to be representative of the immune response to placental parasites: anti-DBL5ε- IgG level correlates with anti-VSAs IgG level and with parity, women with a high level of anti-DBL5ε- IgG at enrolment were shown to more likely present with an uninfected placenta at delivery [13]. Furthermore, monoclonal antibodies inhibiting IE adhesion to placental cryosection are able to recognize recombinant DBL5ε- [8]. Therefore, a refined analysis of the anti-DBL5ε- response in these women was justified.

IgG3 was the only isotype of anti-DBL5ε- IgG showing a significant difference between women with a placenta infected or not, according to parity, thus only the results concerning this subclass is presented here. In primigravidae infected during pregnancy, the level of IgG3 was higher at delivery than at enrolment. This suggests that this IgG subtype is predominantly implicated in the acquisition of immunity against a placental infection, as reported by Megnekou *et al*. [11] and Elliott *et al*. [10] for antibodies to VSAs, and that IgG3 predominantly recognize DBL5ε- among the repertoire of VSAs.

Anti- DBL5ε- IgG3 levels at enrolment increased with parity (p = 0.08), in line with other studies showing that primigravidae have lower levels of IgG against IE surface antigens than multigravidae [15]. This high level of IgG3 in multigravidae may be, at least partially, responsible for the reduced susceptibility to PAM. Primigravidae presenting with a placental infection at delivery, had a lower level of anti-DBL5ε- IgG3 at enrolment, at delivery, and a smaller variation of these antibodies between enrolment and delivery, than primigravidae without placental infection, suggesting that these antibodies could have an impact in preventing placental infections. The levels of IgG3 in 21 Cameroonese primigravidae (enrolled in 1993 in Ebolowa, 160 km south from Yaounde) was analysed according to placental infection (data not shown). The same trend but not significant (probably due to the small sample size) was observed. In term of public health, this protective effect seems important since primigravidae are the most susceptible to PAM consequences. In contrast, in multigravidae, placental infection was not related with the level of anti-DBL5ε- IgG3 at enrolment, but was associated to a higher level of anti-DBL5ε- IgG3 at delivery. In this situation, these antibodies seem to react as a marker of infection rather than as a marker of protection from PAM.

A similar interaction between placental malaria and gravidity was reported for the risk of parasitemia in infancy. Infants born from infected primigravidae have a lower risk of parasitemia than infants born from uninfected primigravidae. Whereas, the risk is reversed in multigravidae, with infants born from infected multigravidae having a higher risk of parasitaemia [16].

Selected DBL3X sequence motifs in VAR2CSA are more likely encountered in parasites from primigravidae, while other motifs are more likely present in multigravidae [9]. A similar mechanism may occur for other VAR2CSA domains, and even other VSAs, with parasites infecting primigravidae and multigravidae expressing different variants. Human are often infected by multiple variants that will compete for host resources, often resulting in one variant emerging among the population [17]. It could be hypothesized that parasites infecting pregnant women commonly express a particular DBL5ε-, conferring a high binding capacity, representing a biological advantage in pregnant women. First-time pregnant women are likely to be infected by this variant, that will be able to outgrow and fastly dominate rarer and less advantaged variants. Those variants that are frequent in the most susceptible group of primigravid women are probably the most virulent and would be of particular interest in the development of efficient vaccine against PAM. High level of protective IgG aquired against common variants during first pregnancies will favour establishment of new and less frequent variants in multigravidae. Thus, the inverted relation observed in this study supports distincts mechanisms according to parity and also probaly to variants. While the protectiveness of anti-DBL5ε- IgG3 subclass in primigravidae is clear, this can rather turn to a marker for infection in multigravidae.

## Conclusion

Results presented here highlight the fact that VAR2CSA possesses immunogenic epitopes that can be of major interest for any vaccination strategy aiming to provide protection to primigravidae which are most at risk. It seems, therefore, important to pursue the sequence analyses of the various DBLs of the VAR2CSA expressed by placental parasites isolated from different parities, in order to determine critical epitopes.

## Authors' contributions

TN, and PD conceived and designed the experiments.

NMM, TL, JG carried out the ELISA experiments.

GC, PD, JG, TN analysed the data.

SS, AS contributed to reagents and materials.

JG, GC, PC, TN wrote the paper.

All authors read and approved the final manuscript.
